# Vitamin D for prevention of sternotomy healing complications: REINFORCE-D trial

**DOI:** 10.1186/s13063-020-04920-z

**Published:** 2020-12-11

**Authors:** Michal Čečrle, Dalibor Černý, Eva Sedláčková, Barbora Míková, Vlasta Dudková, Eva Drncová, Michala Pokusová, Ivo Skalský, Jana Tamášová, Milada Halačová

**Affiliations:** 1 Department of Clinical Pharmacy, Na Homolce Hospital, Prague, Czech Republic; 2 Institute of Pharmacology, 1st Faculty of Medicine, Charles University, Prague, Czech Republic; 3 Department of Cardiac Surgery, Na Homolce Hospital, Prague, Czech Republic; 4 Department of Radiology, Na Homolce Hospital, Prague, Czech Republic; 5 Department of Clinical Biochemistry, Hematology and Immunology, Na Homolce Hospital, Prague, Czech Republic; 6 Hospital Pharmacy, Na Homolce Hospital, Prague, Czech Republic; 7 Department of Medical Physics, Na Homolce Hospital, Prague, Czech Republic; 8 Department of Pharmacology, 2nd Faculty of Medicine, Charles University, Prague, Czech Republic

**Keywords:** Vitamin D, Cholecalciferol, Cardiac surgery, Sternotomy, Healing complications, Trial design, Randomized clinical trial

## Abstract

**Background:**

Most cardiac surgery patients undergo median sternotomy during open heart surgery. Sternotomy healing is an arduous, very complex, and multifactorial process dependent on many independent factors affecting the sternum and the surrounding soft tissues. Complication rates for median sternotomy range from 0.5 to 5%; however, mortality rates from complications are very variable at 7–80%. Low calcidiol concentration below 80 nmol/L results in calcium absorptive impairment and carries a risk of bone loss, which is considered as a risk factor in the sternotomy healing process. The primary objective of this clinical trial is to compare the incidence of all postoperative sternotomy healing complications in two parallel patient groups administered cholecalciferol or placebo. The secondary objectives are focused on general patient recovery process: sternal bone healing grade at the end of the trial, length of hospitalization, number of days spent in the ICU, number of days spent on mechanical lung ventilation, and number of hospital readmissions for sternotomy complications.

**Methods:**

This clinical trial is conducted as monocentric, randomized, double-blind, placebo-controlled, with planned enrollment of 600 patients over 4 years, approximately 300 in the placebo arm and 300 in the treatment arm. Males and females from 18 to 95 years of age who fulfill the indication criteria for undergoing cardiac surgery with median sternotomy can be included in this clinical trial, if they meet the eligibility criteria.

**Discussion:**

REINFORCE-D is the first monocentric trial dividing patients into groups based on serum calcidiol levels, and with dosing based on serum calcidiol levels. This trial may help to open up a wider range of postoperative healing issues.

**Trial registration:**

EU Clinical Trials Register, EUDRA CT No: 2016-002606-39. Registered on September 8, 2016.

## Administrative information

**Table:** 

Title {1}	**Vitamin D for prevention of sternotomy healing complications: REINFORCE-D trial** REINFORCE-D: **Resurgence** **in** **sternotomy** **fortified by vitamin** **D** **supplementation in** **cardiac surgery**
Trial registration {2a and 2b}.	2a EU Clinical Trials Register, EUDRA CT No: 2016-002606-39, registered on September 08, 2016, https://www.clinicaltrialsregister.eu/ctr-search/trial/2016-002606-39/CZ. 2b World Health Organization Trial Registration Data Set – please refer to 2a
Protocol version {3}	The updated protocol is at version 5, on October 2016.
Funding {4}	Supported by Ministry of Health, Czech Republic - conceptual development of research organization (NNH, 00024883 – IG No 168602, IG No 168601) Supported by Charles University in Prague, Czech Republic - grant SVV 260 263.
Author details {5a}	^1^Department of Clinical Pharmacy, Na Homolce Hospital, Prague, Czech Republic ^2^Institute of Pharmacology, 1st Faculty of Medicine, Charles University, Prague, Czech Republic ^3^Department of Cardiac Surgery, Na Homolce Hospital, Prague, Czech Republic ^4^Department of Radiology, Na Homolce Hospital, Prague, Czech Republic ^5^Department of Clinical Biochemistry, Hematology and Immunology, Na Homolce Hospital, Prague, Czech Republic ^6^Hospital Pharmacy, Na Homolce Hospital, Prague, Czech Republic ^7^Department of Medical Physics, Na Homolce Hospital, Prague, Czech Republic ^8^Department of Pharmacology, 2nd Faculty of Medicine, Charles University, Prague, Czech Republic **Corresponding author:** Dalibor Černý, Department of Clinical Pharmacy, Na Homolce Hospital, Roentgenova 37/2, 150 30 Prague 5, Prague, Czech Republic, +420 731 680 765 Email: **dalibor.cerny@homolka.cz**
Name and contact information for the trial sponsor {5b}	Na Homolce Hospital, Roentgenova 37/2, 150 30 Prague 5, Prague, Czech Republic, +420 731 680 765
Role of sponsor {5c}	Sponsor is responsible for trial design; collection, management, analysis, and interpretation of data; writing of the report; and the decision to submit the report for publication.

## Introduction

### Background and rationale {6a}

Vitamin D3 (cholecalciferol) is produced by the human skin from its precursor 7-dehydrocholesterol, or received from diet. Initially, cholecalciferol undergoes 25-alpha hydroxylation in the liver, forming calcidiol. Another hydroxylation takes place primarily in the kidneys, where calcidiol is metabolized to calcitriol by 25-hydroxyvitamin D3-1α-hydroxylase. The crucial physiological effect is to increase serum calcium levels by stimulating renal calcium retention, intestinal calcium resorption, and bone resorption to maintain normocalcemia. Calcitriol predominantly affects bone, tooth, and cartilage mineralization indirectly by regulation of local calcium and phosphate levels; however, there is also direct action on mineralization as calcitriol has genomic and non-genomic effects on cells of mineralizing tissues [1]. Calcitriol binds to the vitamin D nuclear receptor (VDR). VDRs, present in many tissues, are involved in the expression of more than 500 of the total 20,488 genes in the human genome [2]. The 25-OH hydroxyvitamin D (calcidiol) with its elimination halftime of 15 days is the best indicator of the vitamin D saturation state in the body, as it takes into account both vitamin D3 produced endogenously in the skin and exogenous vitamin D2 and D3 from the diet. The definition of individual vitamin D saturation status based on blood calcidiol concentration is controversial [3]. For the purposes of our clinical trial, we have defined it as displayed in Table 1, with a value for serum calcidiol of 80 nmol/L as the low end of the optimal range. Serum calcidiol below this level results in impaired calcium absorption and carries a risk of bone loss and osteoporotic fractures [4, 5].

**Table 1:** Definition of vitamin D saturation status based on serum calcidiol levels

Serum calcidiol concentration in ng/mL and nmol/L, respectively		Vitamin D saturation status—interpretation of values
< 10 ng/mL	< 24 nmol/L	Deficiency
10–20 ng/mL	25–49 nmol/L	Insufficiency
20–30 ng/mL	50–74 nmol/L	Sufficiency
30–50 ng/mL	75–125 nmol/L	Optimal level (considered for this clinical trial)
> 100 ng/mL	250 nmol/L	Risk of toxicity

#### Rationale for cholecalciferol supplementation in cardiac surgery patients

The results of several epidemiological studies suggest a correlation between cardiovascular disease, including cardiovascular mortality, and low calcidiol levels [6]. VDR and 25-hydroxyvitamin D3–1-alpha-hydroxylase have also been found in vascular smooth muscle, endothelium, and cardiomyocytes [7]. Calcitriol controls the proliferation of these cells through genomic and non-genomic effects [8]. There is a growing amount of evidence showing a negative effect of calcidiol deficiency on the cardiovascular system. Calcidiol deficiency leads to cardiac hypertrophy, vascular calcifications, progression of heart failure, and myocardial fibrosis. Low calcidiol blood concentrations are associated with higher morbidity and mortality from cardiovascular disease [9, 10]. A large German study (4418 cardiac surgery patients) evaluated calcidiol concentration in cardiac surgery patients at the time of hospitalization, and found that 38% of patients had deficient blood concentrations of calcidiol (below 30 nmol/L) and an additional 32.3% patients had an insufficient concentration (30–49.9 nmol/L). In these patients, a correlation between low levels of calcidiol and greater postoperative cardiovascular complications was found (higher number of ventilation days, higher number of cardiac and cerebrovascular events; MACE). Patients with optimal calcidiol levels in the range of 75–100 nmol/L showed the lowest number of these events. However, only 7.3% of patients achieved such levels. Patients with insufficient levels of 50–74.9 nmol/L formed an imaginary gray zone of 19.2%, where the number of these events was partially reduced but without statistical significance [11].

During open heart surgery, most patients undergo median sternotomy. Sternotomy is, per se, considered as a fractured sternum. Sternotomy healing is an arduous, very complex, and multifactorial process dependent on many independent factors affecting not only the sternum, but also soft tissues close to the sternum. It has been suggested that low blood calcidiol concentration may be one of these factors, as low calcidiol concentration can lead to osteoporosis and impaired bone healing [4]. Numerous methods have been applied to enhance fracture healing in the setting of osteoporosis [12]. Although the majority of studies in animals support the beneficial effects of cholecalciferol on fracture healing, data from larger randomized trials are still lacking [13]. There is also some evidence of a negative correlation between low calcidiol levels and poor wound healing. The healing of soft tissues adjacent to the sternum is directly dependent on sternal stability. There is also evidence of a positive vitamin D effect on skin wound healing by systemic treatment [14].

#### Sternotomy healing and its complications

Complication rates for median sternotomy range from 0.5 to 5%; however, mortality rates from complications are variable (7–80%). Complications may affect the presternal (cellulitis, sinus tracts, abscess), sternal (osteomyelitis, dehiscence), or retrosternal (mediastinitis, hematoma, abscess) compartments [15]. According to previous studies, superficial complications occurred in 1.1–6.7% of patients, whereas the incidence of deep sternal wound complications ranged from 0.1 to 3.7% [16–18]. In another single-center study with 1279 patients, 76 patients (5.8%) developed sternal wound complications, superficial healing disorders occurred in 43 patients (3.3%), while 33 patients (2.5%) developed deep wound complications [19].

For the purposes of this clinical trial, sternotomy healing complications within the trial are defined as follows:

1. Presternal complications—an infective or non-infective complication affecting the superficial soft tissues occurring at any time during the clinical trial, not affecting the sternum or mediastinum.
2. Sternal complications—an infective or non-infective complication affecting the sternum (resulting in partial or complete sternal dehiscence or instability), occurring at any time during the clinical trial.
3. Retrosternal complications—an infective or non-infective complication affecting the mediastinal tissues occurring at any time during the clinical trial.

The optimal length of the sternum healing is, per se, the subject of debate. According to one study published in 2014, the sternum is not fully recovered after 3 nor after 6 months [20]. In this clinical trial, a 6-month period for evaluation was selected due to the high probability of capturing any sternotomy healing complications related to bones and soft tissues during the trial.

#### Rationale for cholecalciferol choice

There are two main dietary sources of vitamin D: ergocalciferol (vitamin D2) and cholecalciferol (vitamin D3), both of which are converted to one common 25-hydroxyderivative, called calcidiol. Cholecalciferol is more beneficial for supplementation than ergocalciferol. One clinical trial showed that calcidiol serum level increased three times more when using cholecalciferol than ergocalciferol. Thus, ergocalciferol is less effective than vitamin D3. After administration of cholecalciferol, the concentration of calcidiol is maintained for 14 days, while after vitamin D2 there is a rapid decrease after an initial increase [21]. There are other vitamin D derivatives on the pharmaceutical market, such as alpha calcidiol or calcitriol. We elected not to use them in the present clinical trial as we wanted to only use a natural source of vitamin D that is available to humans under physiological conditions. Low levels of calcidiol are associated with higher parathyroid hormone activity and higher bone resorption. Vitamin D concentration below 12 ng/mL (30 nmol/L) also leads to sarcopenia and thus to decreased muscle strength. Cholecalciferol at a preventive dose of 800 IU/day reduces the risk of falling by more than 20% in seniors [22]. For optimal muscle strength in both active and inactive persons, a calcidiol level of at least 20 ng/mL (50 nmol/L) is required. Serum calcidiol concentration above 30 ng/mL (75 nmol/L) correlates with decreased osteoporotic fractures and falls, higher bone density, and improved dental status. Oral cholecalciferol supplementation at doses of 700–800 IU/day reduces the risk of both pelvic fractures and other bone fractures in elderly patients in both outpatient and long-term care settings. An oral dose of vitamin D 400 IU/day is insufficient to prevent fractures [23]. There are trials describing the beneficial effect of cholecalciferol supplementation on bone healing after fractures of various localizations and etiologies [24, 25]. However, there is no specific level of EBM evidence for a defined cholecalciferol dose in the healing of acute bone fractures; generally, 600–4000 IU/day is recommended [26]. Serum calcidiol levels under 49 nmol/L are generally considered as insufficient (details in Table 1). Although the optimal serum calcidiol concentration for both skeletal and non-skeletal health is controversial, in our clinical trial, all non-placebo group patients will be supplemented with cholecalciferol individually to achieve serum calcidiol levels in the range of 75–100 nmol/L at the end of the clinical trial.

Approved indications for cholecalciferol in the Czech Republic are as follows: (1) the prevention and treatment of rickets and osteomalacia in children and adults, and (2) the prevention of rickets in premature newborns [27]. Use of cholecalciferol for other indications related to vitamin D deficiency is considered *off label*.

Another issue is the co-administration of cholecalciferol with calcium and vitamin K2 for the prevention of osteoporosis. Calcium and vitamin D co-supplementation is very common; however, it has shown inconclusive safety data regarding to increased incidence of various cardiovascular complications [28]. In this clinical trial, it has elected not to co-administer calcium to our patients. Subjects in this trial will be advised to maintain a balanced diet including dairy products and other calcium sources. Cholecalciferol co-supplementation with the vitamin K2 subtype menatetrenone (MK4) may be beneficial, as menatetrenone plays a key role in bone calcium balance [29, 30]. However, we elected not to follow this strategy, as vitamin K2 MK4 as well as vitamin K1 (phylloquinone) may interfere with warfarin pharmacotherapy. Warfarin may be unavoidable in some trial subjects (e.g., patients with mechanical valve prostheses), and its potential failure could be fatal for these patients.

#### Rationale for cholecalciferol dosing and timing

We elected to administer the trial medication (IMP) once per week due to better patient compliance. Moreover, we introduced a system which automatically sends SMS reminders every week at the same time.

There are a number of different recommendations in the literature with regard to how to supplement vitamin D. There is the extensive German systematic review from 2014, which evaluated different dosing schedules from 1975 to 2013 in a total of 144 studies on 11,500 patients for an average of 274 days, i.e., 9 months. According to this review, supplementation is dependent on patient weight and age, and dosing schedules were as follows [31]:

- For a 75-kg patient, 30 years of age, a dose of 2520 IU/day increased the calcidiol value by 50 nmol/L (or from 25 to 75 nmol/L) for 274 days, corresponding to a total cumulative dose of 690,480 IU vitamin D (1380 drops; 38 drops/week).
- For a 75-kg patient, 70 years of age, a dose of 1460 IU/day increased the calcidiol value by 50 nmol/L (or from 25 to 75 nmol/L) for 274 days, corresponding to a total cumulative dose of 400,040 IU vitamin D (800 drops; 22 drops/week).

Based on the above data, we created our own scheme that would be as simple as possible for patients and nurses during the hospital stay and would lead to optimal vitamin D saturation according to the degree of initial deficiency. The total duration of supplementation will last 182 days (6 months) and will be implemented in two phases as follows:

1. *Initial single bolus 25,000 IU (50 drops)*

All the trial patients with calcidiol levels between 0 and 75 nmol/L will receive a uniform bolus of 50 drops (i.e., 25,000 IU) of IMP at time 0 before cardiac surgery. This bolus will provide at least a minimum baseline saturation for all subjects before cardiac surgery. It has been shown repeatedly that administration of an oral bolus of up to 100 drops (50,000 IU) is advantageous and safe. Some authors have claimed that a single bolus of 100,000 IU is safe [32]. Moreover, in a systematic review, a bolus of 300,000 IU was reported as safe and well tolerated [33, 34]. These data suggest that a bolus of 50 drops will be a safe dose of vitamin D for the whole clinical trial population.

2) *Continued dosing schedule*

To calculate the optimal dosing for three patient cohorts in this clinical trial, we followed the recommendations from the review mentioned above, also incorporating information from another review [31, 35].

After the initial bolus, dosing will continue for 26 weeks in weekly intervals, starting from the day that the patient received the first preoperative bolus:

- Patient cohort with vitamin D deficiency (calcidiol 0–24 nmol/L) will receive 40 drops per week (2986 IU/day). We expect a 75-nmol/L increase in calcidiol blood concentration in the non-placebo group. The total cumulative vitamin D3 dose is 545,000 IU per 6 months.
- Patient cohort with vitamin D insufficiency (calcidiol 25–49 nmol/L) will receive 25 drops per week (1917 IU/day). We expect a 50-nmol/L increase in calcidiol blood concentration in the non-placebo group. The total cumulative vitamin D3 dose is 350,000 IU per 6 months.
- Patient cohort with vitamin D sufficiency (calcidiol level 50–75 nmol/L) will receive 10 drops per week (849 IU/day). We expect a 25-nmol/L increase in calcidiol blood concentration in the non-placebo group. The total cumulative vitamin D3 dose is 155,000 IU per 6 months.

The placebo subjects in each group will receive the same number of drops of inert vehicle (*Triglycerida saturata media*), assuming that the number of drops of placebo does not play a role in placebo efficacy.

### Objectives {7}

The primary objective of this clinical trial is to compare the incidence of all postoperative sternotomy healing complications (presternal, sternal, and retrosternal) in two parallel patient groups with cholecalciferol or placebo treatment.

As a primary hypothesis, it has been expected that cholecalciferol supplementation in heart surgery patients with deficient or insufficient blood calcidiol concentration (based on initial calcidiol blood concentration) will show a reduced incidence of postoperative sternotomy healing complications within the 6-month period after sternotomy.

The secondary objectives are focused on general patient recovery: sternal bone healing grade at the end of the trial, length of hospitalization, number of days spent in the ICU, number of days spent on mechanical ventilation, and number of hospital readmissions for sternotomy complications.

### Trial design {8}

This clinical trial is conducted as a monocentric, randomized, double-blind, placebo-controlled trial, with the goal to enroll 600 patients over 4 years: approximately 300 subjects in the placebo arm and 300 in the treatment arm. Males and females aged 18 to 95 years who fulfill the indication criteria for undergoing cardiac surgery with sternotomy may be included in this clinical trial, if they meet the eligibility criteria (as shown in Table 2).

**Table 2:** Patient eligibility criteria

Inclusion criteria	Exclusion criteria
Patient:	Patient:
• Undergoing cardiac surgery with sternotomy	• With known hyperparathyroidism
• With baseline calcium, phosphate, and alkaline phosphatase values in physiological ranges	• Treated or supplemented by vitamin D or its derivatives (alfacalcifediol, calcitriol, paricalcitol) including ergocalciferol (vitamin D2) when used regularly for 3 months before entering the trial
• With sufficient ability to understand and cooperate in planned medical examinations	• Undergoing repeated sternotomy
• Czech or Slovak speaker with permanent residence and citizenship in the Czech Republic	• With chronic renal insufficiency associated with osteodystrophy (hyperphosphatemia, hypocalcemia)
• With initial calcidiol levels less than 75 nmol/L	• With severe hepatic impairment associated with cirrhosis, Child-Pugh score B and C
• With signed informed consent to enter the trial	• With known metabolic bone disease
• Male or female between 18 and 95 years of age	• With cancer associated with primary or secondary risk of bone damage
	• With known immunodeficiency
	• With short bowel syndrome or other malabsorption syndrome that limits absorption of lipophilic vitamins
	• With long-term cardioactive glycoside and/or thiazide diuretic treatment that cannot be replaced with alternative treatment during cardiac surgery hospitalization
	• Prior to the planned radiotherapy affecting the body skeleton
	• With known allergic reaction or intolerance to IMP
	• With long-term teriparatide treatment or other parathyroid hormone
	• With long-term bile acid sequestrant treatment which cannot be replaced by any other treatment during cardiac surgery hospitalization
	• With long-term corticosteroid treatment or other immunosuppressive drugs
	• With another medical condition which the investigator considers a risk for the patient during the trial
	• In custody or imprisonment or receiving healthcare without their consent for the purpose of protecting the public interest or protecting the life and health of the individual by law
	• Pregnant, nursing, or planning to become pregnant during the trial
	• Participating in another clinical trial

## Methods: participants, interventions, and outcomes

### Study setting {9}

The trial is monocentric, based at the Cardiac Surgery Department of Na Homolce Hospital, Prague, Czech Republic.

### Eligibility criteria {10}

All the eligibility criteria are summarized in Table 2.

All medical interventions (except of blood sampling) are performed by clinical trial examiner (medical doctor).

### Who will take informed consent? {26a}

The main examiner (medical doctor) suggests patient participation in the trial by oral interview. Patients fulfilling the inclusion criteria and not meeting the exclusion criteria will be offered participation in the trial when the decision for sternotomy is achieved.

To control selection bias, a prospective database of patients not participating in the study will be maintained at each attending hospital during the study period.

### Additional consent provisions for collection and use of participant data and biological specimens {26b}

There are no special provisions for the collection and use of participant data and biological specimens. There are no ancillary studies. The biological material is disposed of in accordance with the relevant hospital regulations.

## Interventions

### Explanation for the choice of comparators {6b}

Pharmaceutical-quality saturated triglycerides (*Triglycerida saturata media*) will be used as a placebo, as an identical compounding vehicle is used in the original medicinal product Vigantol®.

### Intervention description {11a}

The only therapeutic intervention is cholecalciferol/placebo administration. The total duration of supplementation will be 182 days (6 months). It will run in two stages:

1. *A 50-drop bolus*. This initial bolus provides at least minimal initial saturation in all patients prior to cardiac surgery.
2. *Continued dosing schedule.* Patients with 0–24 nmol/L calcidiol will receive 40 drops per week, those with 25–49 nmol/L will receive 25 drops per week, and those with calcidiol 50–75 nmol/L will receive 10 drops per week.

The rationale for dosing and length of supplementation is described above in the “Introduction” section.

### Criteria for discontinuing or modifying allocated interventions {11b}

No interventions or drug dose changes will be made during the trial. If there is any problem related to the clinical trial, patient participation in clinical trial will be terminated. The decision for termination can be made from both sides:

1. Patient decision
  A trial participant may leave the trial at any time without providing a reason.
2. Examiner decision
  Due to the fact that the clinical trial is based on the administration of cholecalciferol or placebo, termination of the clinical trial by the investigator is very unlikely. Nevertheless, in rare circumstances described below, the examiner may decide to exclude a subject from the trial due to preventive reasons.

Criteria for the clinical trial discontinuing by examiner:

- Cholecalciferol intolerance
- Hypercalcemia—serum total calcium concentration higher than 2.75 mmol/L, measured in at least one of the scheduled laboratory tests
- Hyperphosphatemia—concentration of total phosphorus in the serum above 1.61 mmol/L, measured in at least two of the scheduled laboratory tests
- An increased serum alkaline phosphatase activity two times higher than the physiological range, measured in at least two of the scheduled laboratory tests
- A high level of calcidiol above 200 nmol/L, measured in a targeted manner in case of suspected overdose at any time during CT
- The need to use prohibited medication
- Pregnancy

Interruption or termination of CT for other reasons is always subject to the judgment of the examiner (medical doctor), but due to its design is not expected.

In both decisions of this participant’s termination, trial subject will be asked to do the following:

- To return the rest of the IMP at the hospital Cardiac Surgery Department
- To be medically examined within 14 days from the medication withdrawal (calcidiol concentration, calcium concentration)—these examinations can also be requested by a general practitioner in the place of trial subject residence

### Strategies to improve adherence to interventions {11c}

Automatic SMS reminders will be introduced. Every week, the patient will receive a message with specific individualized dosing information. The reminder of the IMP will be returned to the examiner.

### Relevant concomitant care permitted or prohibited during the trial {11d}

Participants should record and report the number of days spent actively sunbathing, if at least half of the body is exposed to the sun.

#### Concomitant medication permitted during clinical trials

With the exception of medication that is expressly prohibited, all medication is allowed without restriction.

#### Prohibited concomitant medication during the clinical trial

1. Systemic immunosuppressive pharmacotherapy (including corticotherapy) more than 14 days in duration or exceeding the length of hospitalization (if the actual duration of treatment could not be determined), or local corticotherapy applied at the site of wound healing more than 5 days duration.
2. Systemic pharmacotherapy with digitalis cardioactive glycosides at any dosage if not possible to ensure regular monitoring of calcium with a minimum measurement interval of once per week, always before the administration of IMP.
3. Systemic pharmacotherapy with hydrochlorothiazide at a dose higher than 12.5 mg/day if not possible to ensure regular monitoring of calcium with a minimum measurement interval once per week, always before the administration of IMP.
4. Chronic treatment with teriparatide.
5. Chronic treatment with bile acid sequestrant.
6. Chronic treatment with vitamin D or its derivatives (alphacalcifediol, calcitriol, paricalcitol) including ergocalciferol (vitamin D2) in addition to IMP.
7. Administration of any medicinal products or food supplements containing any calcium salt in any dosage if not possible to ensure regular monitoring of calcium with a minimum measurement interval of once every 2 months, always before the administration IMP.

### Provisions for post-trial care {30}

There are no provisions included. Entering the trial is voluntary. The trial is not sponsored by any pharmaceutical company. The trail will be paid from public funds provided for Na Homolce Hospital. Therefore, it is not dependent on the pharmaceutical industry, but is conceived as low-cost. Therefore, there is no financial reward for participation in the trial, nor is there any other compensation for the subject in connection with participation in the trial (e.g., reimbursement of travel expenses).

### Outcomes {12}

#### Efficacy outcomes

##### Primary endpoint

1. The number of all postoperative sternotomy healing complications in two parallel patient groups with cholecalciferol or placebo treatment (calculated incidence in %).
  Patients with sternotomy healing complication have to fulfill the following 2 conditions that can occur at any time during the clinical trial period (evaluation time points: 6 weeks and 6 months following sternotomy):
  - Sternotomy soft tissue dehiscence (evaluated by medical examination) by visual inspection, palpation, and auscultation in the surgical area of sternotomy.
  - Sternal bone crepitation (evaluated by medical examination) by visual inspection, palpation, and auscultation in the surgical area of sternotomy.
  Patients not fulfilling both conditions will be considered without sternotomy healing complications.

##### Secondary endpoints

1. Sternal bone healing grade at the end of the trial evaluated by computed tomography using a 6-point scale according to Gregory S. Stacy [20], evaluation made by a certified radiologist (evaluation time point: 6th month).
2. Total number of intensive care unit (ICU) days spent with mechanical ventilation during the original (first) heart surgery hospitalization, obtained from patient records in the hospital information system (evaluation time point: hospitalization discharge).
3. Total number of ICU days during the original (first) heart surgery hospitalization, obtained from the hospital information system (evaluation time point: hospitalization discharge).
4. Number of hospital readmissions for sternotomy complications within the clinical trial duration obtained from patient records in the hospital information system (evaluation time point: 6th month).
5. Total number of treatment days for pericardial and/or pleural effusions during the original (first) heart surgery hospitalization and/or re-hospitalizations, obtained from patient records in the hospital information system.

#### Safety outcomes

1. Safety endpoints related to impaired biochemical examinations, calculated incidence in two parallel patient groups with cholecalciferol or placebo treatment:
  - Serum calcium concentration out of physiological range (values within the range 2.05–2.9 mmol/L considered physiological)
  - Alkaline phosphatase activity out of physiological range (values within the range 0.66–2.2 μcat/L considered physiological)
  - Serum phosphate concentration out of physiological range (values within the range 0.65–1.61 mmol/L considered physiological)
2. Endpoints related to non-serious adverse effects, calculated incidence in two parallel patient groups with cholecalciferol or placebo treatment:
  - Constipation
  - Loss of appetite
  - Nausea
  - Other non-serious adverse effect
3. Endpoints related to serious adverse events, calculated incidence in two parallel patient groups with cholecalciferol or placebo treatment:
  - Death
  - Life-threatening
  - Hospitalization (initial or prolonged)
  - Disability or permanent damage
  - Required intervention to prevent permanent impairment or damage (devices)
  - Important medical event

Efficacy and safety endpoint evaluation timetable is shown in Table 3.

**Table 3:** Efficacy and safety endpoint evaluation timetable

Week	Examination of the subject by the investigator	Laboratory examination of the subject	Evaluation of subject’s quality of life	Imaging examination of the subject
**0**	**Initial interview with the patient + initial medical examination + decision to offer clinical trial participation to the patient**	**Initial complete biochemical examination including monitored parameters (calcium, phosphate, calcidiol, and ALP)**	**Acquaintance with the trial + provision of signed informed consent**	
**1**		**Continuous determination of several biochemical parameters during hospitalization: serum calcium (minimally every other day), ALP values (minimally once during hospitalization), and serum phosphate (minimally once during hospitalization)**		
**2**	**Examination of the surgical wound before hospital discharge**	**Laboratory tests before discharge**		
**6**	**Continuous examination of surgical wound**	**Continuous evaluation of serum calcium, ALP, and phosphate**		
**26**	**Exit examination of the surgical wound**	**(1) Final lab. test of serum calcium, ALP, and phosphate; (2) calcidiol level; (3) doctor’s entry in Questionnaire No. 1**	**Final evaluation of quality of life according to Questionnaire No. 2**	**Computed tomography of the chest**

### Participant timeline {13}

#### Enrollment

The main examiner (anesthesiology specialist) suggests patient participation in the trial by oral interview. Patients fulfilling the inclusion criteria and not meeting the exclusion criteria will be offered the participation in the trial when a decision of a sternotomy by surgeon is achieved.

#### Assessments and visits

Daily visits to the participants will be conducted by a cardiac surgeon while in the hospital for whole period of hospitalization to evaluate relevant outcome measures. The cardiac surgeon will examine the sternotomy for possible complications. All the biochemical examinations are indicated by any medical doctor in the cardiac surgery ward (anesthesiology specialist, cardiology specialist, or cardiac surgeon). The last evaluation will be done on the day of discharge, then after in the sixth week and in the sixth month after cardiac surgery (including sternotomy).

Participant timeline is displayed in Table 4.

**Table 4:**
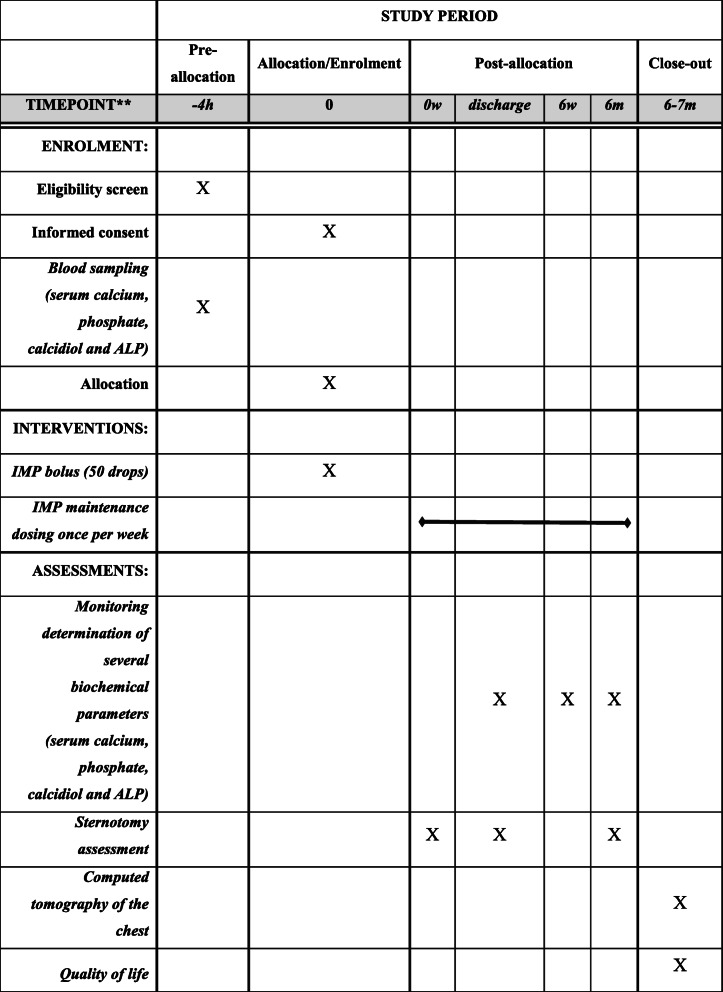
Participant timeline in REINFORCE-D clinical trial

### Sample size {14}

To determine the optimal trial patient population, a sample size calculation was made. This calculation is based on the estimated incidence of sternotomy healing complications, which is typically around 5% at our cardiac surgery center (unpublished internal data). This is in agreement with data published previously. If we assume the same incidence during the trial, and considering vitamin D supplementation, we expect an incidence of roughly 1%. Thus, 284 patients are needed in the placebo arm and 284 patients in the treated arm, considering a one-sided statistical alternative. We plan to enroll approximately 300 patients in each arm, altogether 600 patients.

MedCalc software (version 17.8.6) was used to calculate sample size. The following input data was used for sample size calculation:

- *p*1 = 0.05, incidence of event occurrence (sternotomy complications) in the placebo arm
- *p*2 = 0.01, incidence of event occurrence (sternotomy complications) in the treatment arm (cholecalciferol)
- α = 0.05, probability of type I error
- β = 0.2, probability of type II error

### Recruitment {15}

Males and females 18 to 95 years of age who fulfill the indication criteria for undergoing cardiac surgery by a standardized scoring system (EUROSCORE) can participate in our clinical trial (CT), if they meet the eligibility criteria below (see Table 2). Subjects that are pregnant, breastfeeding, or planning to become pregnant during CT will be excluded. If a subject becomes pregnant during the CT, they will be terminated from the CT medication and will be removed from CT. However, due to the character of the clinical trial and the medication used, it will not be necessary to require women to use contraceptives.

**Fig. 1:**
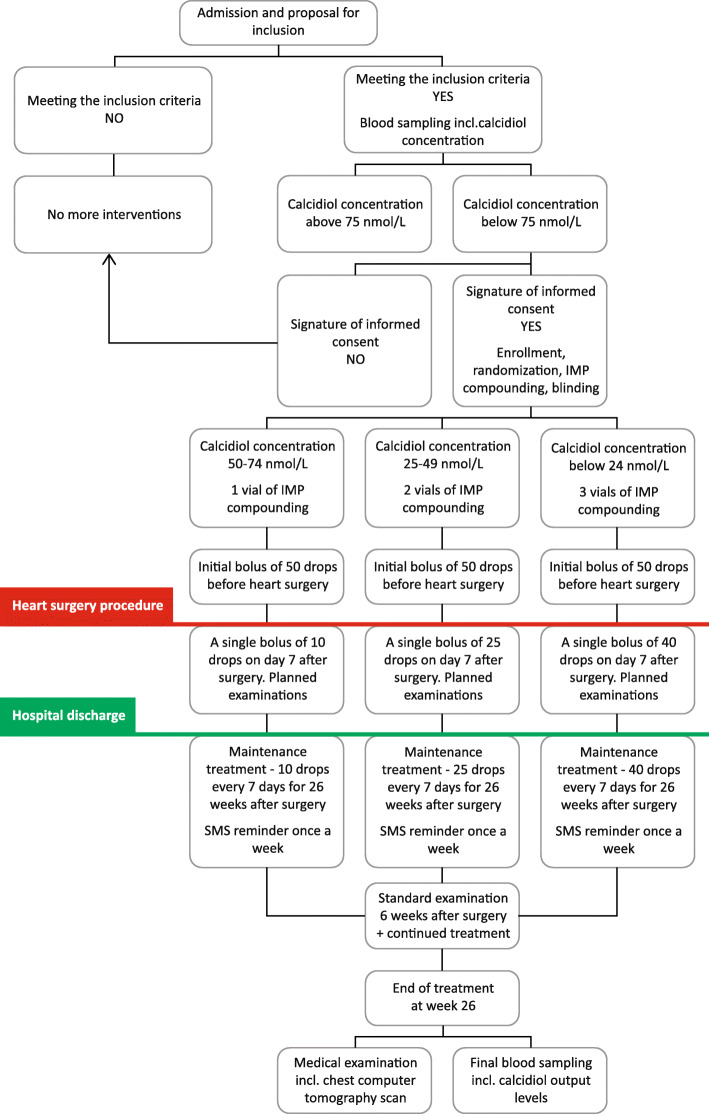
Scheme of clinical trial. Patient who is indicated for heart surgery procedure undergoing sternotomy and its initial serum calcidiol concentration is below 75 nmol/L; he/she becomes an object for enrollment into the CT. If the patient fulfills the inclusion criteria and is not meeting the exclusion criteria at the same time, he/she is offered to participate in the trial. If the patient agrees with the trial participation and signs an informed consent, he/she is randomized

Patients will be admitted to the CT independent of age, gender, race, and type of surgery including sternotomy. Subsequently, initial calcidiol blood concentration will be evaluated for each proposed patient. If the calcidiol blood concentration is below 75 nmol/L, the patient will be offered participation in the CT. By providing signed, informed consent, the patient will become a subject of CT assessment, will receive a CT identification number, and will be subsequently randomized (see Fig. 1).

To control for selection bias, a prospective database of patients undergoing sternotomy and not participating in the trial will be maintained at each site during the trial period.

## Assignment of interventions: allocation

### Sequence generation {16a}

Sequence allocation from number 1 up to number 600 was done using R Core Team randomization software (version 2015) with the accompanying information of whether the respective subject receives a placebo or cholecalciferol. The probability that respective identification number is assigned to placebo or cholecalciferol was set within the ratio of 1:1 (in blocks) to ensure approximately even distribution of both compounded investigational medicinal products (placebo/cholecalciferol) in both CT arms.

### Concealment mechanism {16b}

Items in this list have been individually sealed in randomization envelopes labeled with the identification numbers (1–600) on the front side. The sealed envelopes have been placed in a special room unavailable to those who enroll participants or assign interventions. No other restrictions have been used.

### Implementation {16c}

Sequence allocation has been carried out by a certified independent statistician. Patients are enrolled by a clinical trial examiner (medical doctor) and also assigned to interventions. An independent clinical pharmacist (not blinded) receives information about the newly enrolled patient, unseals the new envelope with the respective patient CT number, and gives the compounding instructions for investigational medication to the compounding pharmacist.

Patient randomization is performed by unsealing the randomization envelope labeled with the appropriate identification number at the time when patient is accepted into the CT and has provided signed, information consent (see Fig. 1). Replacement of previously randomized patients by new patients is not permitted during the CT.

## Assignment of interventions: blinding

### Who will be blinded {17a}

Our clinical trial is conducted as double-blind, so neither the investigator and his team (care providers, outcome assessors) nor the trial participants know whether they are using cholecalciferol or placebo, but they know all clinical data about the trial participants.

Only one independent person (clinical pharmacist), who provides instructions regarding the IMP compounding, is unblinded. The pharmacist is only unblinded to the subject identification number and code of the IMP, but otherwise is blinded to clinical data and the patient’s name.

The blinding information is covered in blinding envelopes created by the pharmacist. The blinding envelope contains 4 details: date of IMP compounding, type of IMP (placebo/cholecalciferol), and batch and amount of IMP.

All personal patient data are blinded for data analyses.

### Procedure for unblinding if needed {17b}

Unblinding is possible in emergent clinical situations by opening the sealed envelope with drug code and blinding information inside, which will be available to medical doctors in the cardiac surgery ward 24 h per day. If the respective sealed envelope is opened (unblinded) for any reason, the patient will automatically be dismissed from the clinical trial.

## Data collection and management

### Plans for assessment and collection of outcomes {18a}

Different outcome investigators are responsible for data collection and assessment (trial examiners, trial clinical biochemist, trial clinical radiologist, trial pharmacists, and trial administrative workers).

There are two electronic databases: Database No. 1 (central trial register) with access for all trial workers except the pharmacist, and Database No. 2 (information about IMP content) with an access restricted to the pharmacist and independent clinical pharmacist.

Trial data from laboratory tests will be copied from hospital patient medical records and stored in one database (Database No. 1) at the end of the clinical trial.

Two paper questionnaires will be used: Questionnaire No. 1 for medical doctor reporting clinical findings and Questionnaire No. 2 for the trial participants. Patient questionnaires and medical assessment questionnaires will primarily be hard copy (paper version), and the data from them will be manually transferred to the central electronic database.

The main clinical trial coordinator is also responsible for clinical trial monitoring.

### Plans to promote participant retention and complete follow-up {18b}

There are no extra plans to promote participant retention. Trial participants have no financial reward for participating in the clinical trial. Automatic SMS reminders for trial medication and all medical appointments are introduced. Patients will return completed patient questionnaires (confirming all medication administrations) at the end of the clinical trial. Complete follow-up will be made by the clinical trial coordinator by telephone.

### Data management {19}

Data are recorded to the trial database and partially to the participant’s protocol (paper form), stored in the corresponding author’s office.

### Confidentiality {27}

Trial data are collected in a shared trial database. The authors of the trial have access to this database only. The database can only be opened via the hospital local network.

### Plans for collection, laboratory evaluation, and storage of biological specimens for genetic or molecular analysis in this trial/future use {33}

All biological specimens are collected and stored according to standard GCP and GLP guidelines. No future use in ancillary studies is planned; therefore, no long-term storage is necessary.

## Statistical methods

### Statistical methods for primary and secondary outcomes {20a}

In the statistical analysis of the primary endpoint, it will be necessary to ascertain whether the incidence of all sternotomy complications (6 months follow-up) differs significantly between the treatment and placebo groups. Data will be expressed as number of patients with or without sternotomy healing complications in the placebo/cholecalciferol groups. The chi-square test will be used for this analysis.

Data of all secondary endpoints will be expressed as arithmetic average ± standard deviation. To analyze these data, a two-group *t* test will be used.

This clinical trial assumes a significance level of 0.05. The software MedCalc (version 17.8.6.) will be used for statistical calculations.

### Interim analyses {21b}

The only trial intervention is based on vitamin D supplementation, so there is no presupposition for harmful effects on trial participants; therefore, no interim analyses and termination decision-making are planned within the trial period.

Rules for terminating trial participation:

1. Decision by the trial subject
  Any subject may elect to withdraw from the clinical trial at any time, without providing a reason. In the case of this decision, the participant will be asked to:
  Return the rest of the IMP to Na Homolce Hospital, Cardiac Surgery.
  Appear within 14 days from the announcement of withdrawal from the clinical trial at the Department of Cardiac Surgery for evaluation of serum calcidiol and calcium (these examinations may also be requested by a general practitioner at the place of the participant’s residence).
2. Decision by the examiner
  Due to the fact that the clinical trial is based on the administration of physiological vitamin D or placebo, termination of the trial on the part of the investigator is very unlikely. Nevertheless, in the rare circumstances described below, the examiner may elect to exclude a participant from the trial due to preventive reasons.

Criteria for CT interruption by examiners:

- IMP intolerance
- Hypercalcemia—serum total calcium concentration higher than 2.75 mmol/L, measured in at least one of the prescribed examinations
- Hyperphosphatemia—concentration of total serum phosphorus above 1.61 mmol/L, measured in at least two of the prescribed examinations
- Increased serum alkaline phosphatase activity above twice the reference range, measured in at least two of the prescribed tests
- A high level of calcidiol above 200 nmol/L, measured in a targeted manner in case of suspected overdose at any time during the trial
- The need to use unauthorized medication
- Pregnancy

Interruption or termination of the trial for other reasons is always subject to appraisal of the examining physician, but due to the trial character and design is not expected.

### Methods for additional analyses (e.g., subgroup analyses) {20b}

Additional subgroup analyses are planned of the primary endpoint in groups with different initial calcidiol concentrations. Three subgroups will be evaluated: 1st with initial calcidiol concentration ≤ 25 nmol/L, 2nd with initial calcidiol concentration > 25 nmol/L and ≤ 50 nmol/L, and 3rd with initial calcidiol concentration > 50 nmol/L. The chi-square test will be used for this analysis.

Another subgroup analysis of the primary endpoint will be focused on sternotomy complication type: presternal, sternal, and retrosternal.

### Methods in analysis to handle protocol non-adherence and any statistical methods to handle missing data {20c}

Any missing data will be explained. Multiple imputations of missing outcome data will be used for sensitivity analyses.

If there are missing data regarding to primary endpoints, the trial participant will be excluded from the final trial evaluation.

If there are missing data from the secondary outcomes, the isolated missing value will be not used for the final trial evaluation.

### Plans to give access to the full protocol, participant-level data, and statistical code {31c}

There will be no public access to the full protocol, participant-level dataset, and statistical code. The datasets analyzed during the current trial are available from the corresponding author on reasonable request.

## Oversight and monitoring

### Composition of the coordinating center and trial steering committee {5d}

There is no trial steering committee.

### Composition of the data monitoring committee, its role and reporting structure {21a}

Data monitoring committee is not a part of the trial.

### Adverse event reporting and harms {22}

Adverse events are reported in the database and reported to the Czech Institute for Drug Control (SUKL).

### Frequency and plans for auditing trial conduct {23}

This clinical trial is continuously monitored by the coordinator. No extra auditing is necessary, because this trial is monocentric.

The Czech Institute for Drug Control could perform an audit at any time.

### Plans for communicating important protocol amendments to relevant parties (e.g., trial participants, ethical committees) {25}

Important protocol modifications will be communicated with the Local Ethics Committee of Hospital Na Homolce by amendments. All modifications will also be registered at Clinical Trials and reported to the national drug regulatory authority.

### Dissemination plans {31a}

The protocol of the trial will be published during the trial. The results concerning the primary and secondary endpoints will be processed during the 2-year follow-up after the last included patient. The results will be published in an open access journal in the following 5 years.

## Discussion

Special task (not discussed above) is radiation dose of patients during clinical trial examination of mediastinum.

In order to comply with Czech law 307/2002, Section 61 (paragraph 1), an optimization study of radiation exposure of patients irradiated in accordance with the internal regulations of Na Homolce Hospital was performed. In the absence of real data, it was necessary to approximate the situation by a similar examination. After consultation with a medical physicist, a computed tomography scan of the mediastinum was considered for data collection and subsequent calculation of the expected radiation load of the patient for 1 examination. The data was collected on the same X-ray equipment that was considered for the study itself.

X-ray equipment used: Computed Tomography Somatom Definition Flash (with the possibility to use a dose saving system—Saphire)

Amount of data used: 17

**Table:** 

	**DLP [mGy·cm]**
**Average**	354.4

This is a value that is consistent with the value given in the forthcoming national radiological standards for chest examination (500 mGy·cm).

In order to determine the radiation load of the patient, the average value of the directly measurable quantity, the product of the dose, and the length of the scanned area (DLP) obtained in this way must be converted to the quantity intended for radiation load evaluation (effective dose—*E*). This can be achieved by recalculating with tabulated conversion factors for the conversion of DLP to *E*. For the chest area, this conversion factor is 0.017 mSv·mGy^−1^·cm^−1^.

It follows that:

$$ E={\mathrm{DLP}}_{\mathrm{avg}}\cdotp 0.017=354.4\cdotp 0.017=6.02\ \mathrm{mSv} $$

This value of radiation load corresponds to 2 years of natural background radiation in our country or half a year on the beach in Brazil. However, given the expected diagnostic information, it is unlikely that this method could be replaced by another equally effective method in terms of recovery of diagnostic information. Replacing the considered modality with a modality such as skiagraphy would result in a lower radiation load, but at the same time, all data from other directions (angles) would be lost, which is not the case if computed tomography (using 3D reconstruction models) is used.

## Trial status

This trial is still ongoing; patient recruitment is not yet complete. The updated protocol is at version 5, on October 2016. The first patient was included on 4 January 2017. Recruitment by the investigating department is planned to continue until 31 December 2020, and the trial period will end in June 2021.

## Abbreviations

ALP: Alkaline phosphatase
CT: Clinical trial
DLP: Dose and length product
EBM: Evidence based medicine
ED: Effective dose
GCP: Good Clinical Practice
GLP: Good Laboratory Practice
IU: International unit
ICU: Intensive care unit
IMP: Investigational Medical Product
MACE: Major adverse cardiovascular events
SmPC: Summary of Product Characteristics
SUKL: State Institute for Drug Control – Czech drug regulatory authority
VDR: Vitamin D receptor
